# Total phenolic and flavonoid content and antibacterial activity of *Punica granatum* L. var. *pleniflora* flowers (Golnar) against bacterial strains causing foodborne diseases

**DOI:** 10.1186/s12906-015-0887-x

**Published:** 2015-10-15

**Authors:** Arash Mahboubi, Jinous Asgarpanah, Parisa Nosrati Sadaghiyani, Mehrdad Faizi

**Affiliations:** Department of Pharmaceutics, School of Pharmacy, Shahid Beheshti University of Medical Sciences, 2660, Vali-Asr Ave, Tehran, 141556153 Iran; Young Researchers and Elite Club, Pharmaceutical Sciences Branch, Islamic Azad University, Tehran, Iran; Department of Pharmacognosy, Faculty of Pharmacy, Pharmaceutical Sciences Branch, Islamic Azad University, (IAUPS), Tehran, Iran; Department of Pharmacology and Toxicology, School of Pharmacy, Shahid Beheshti University of Medical Sciences, 2660, Vali-Asr Ave, Tehran, 141556153 Iran

**Keywords:** Flavonoid, Minimum inhibitory concentration, Minimum bactericidal concentration, Foodborne, Golnar

## Abstract

**Background:**

Flowers of *Punica granatum* L. (Punicaceae) var. *pleniflora,* known as “Golnar” in Iranian traditional medicine have been used for the prevention and treatment of foodborne diseases. In this study, antibacterial activities of ethanol extract of Golnar and its fractions were scientifically evaluated against bacteria causing foodborne diseases including *Staphylococcus aureus, Bacillus cereus*, *Listeria monocytogenes*, *Escherichia coli*, *Shigella dysantriae,* and *Salmonella typhi.* The total phenolic and flavonoid contents of the extract and its fractions were also determined.

**Methods:**

The antibacterial effect of the ethanol extract and its fractions were primarily evaluated by agar well diffusion and their MIC and MBC were determined by broth macro dilution method. The total phenolic and flavonoid contents of the extract and its fractions were measured based on gallic acid and rutin equivalents (GAE and RE), respectively.

**Results:**

After evaluation of total phenolic and flavonoid content the chloroform fraction showed the lowest phenolic and flavonoid contents (3.8 mg GAE/g and 1.1 mg RE/g respectively) and the methanol fraction showed the highest phenolic and flavonoid contents (18.1 mg GEA/g and 3.3 mg RE/g respectively). The total phenolic and flavonoid content was positively associated with the antibacterial activities of the fractions with chloroform extract exhibiting lowest antibacterial activity against *E. coli* (MIC 25 mg/ml) and the methanol fraction exhibiting the highest antibacterial effect against *S. aureus* (MIC 0.19 mg/ml).

**Conclusion:**

Golnar extract showed antibacterial activity against both Gram positive and Gram negative bacteria causing food poisoning. Therefore, the extract can be used for prevention or treatment of foodborne diseases or as preservative in the food industry.

The methanol fraction with the highest phenolic and flavonoid content showed the highest antibacterial effect. This indicates that the phenolic and flavonoid compounds in the extract can be responsible for the effect.

## Background

Foodborne diseases are taking thousands of lives every year. Forty-five million people become food poisoned, 128,000 people are hospitalized, and it takes 3000 lives in the USA annually [1]. Therefore, food safety is an important issue for consumers and the food industry. The food industry is now following the consumer opinions for safer additives, especially natural preservatives [2, 3]. Discovering plants and their active ingredients to prevent or cure infections, including foodborne diseases, could be a great achievement [3]. There are at least two important reasons for clinical scientists to get interested in the potential antimicrobial effects of plants. The first reason is the increasing resistance of bacteria to common antimicrobial agents and the second is the unpleasant side effects of synthetic antimicrobial agents. More than that, the antibacterial effects of various medicinal plants are being investigated due to the toxicological concerns associated with the synthetic antioxidants and preservatives [4, 5].

*P. granatum* L. (Punicaceae) is widely cultivated in Iran and has an extensive geographical distribution from Iran to Himalayas in northern India [6]. *Punica granatum* var. *pleniflora* is endemic to Iran and grows as a bush or shrub 2–5 m in height. The flowers are odorless but colorful red or reddish, 3.5 to 7 cm in length, campanulate or cylindrical. Flowers are two types. One of them is the fertilized with large and long- styled, long-stamened, and colorful flowers. The other is the unfertilized with smaller, barren and short- styled, short-stamened flowers, in which the stigma is far below the anthers [7, 8]. The unfertilized flowers are commonly known as “Golnar” in Iranian traditional and complementary medicine [9–11].

Golnar has been extensively used in Iranian traditional medicine as an astringent, haemostatic, and antimicrobial agent. It has also been used as a treatment for bronchitis, diarrhea, digestive problems, infected wounds, and diabetes [9–11]. *P. granatum* fruit (pomegranate) and its pericarp are known to have high molecular weight phenolic compounds including condensed tannins and hydrolysable tannins [12–14]. Several studies indicate that *P. granatum* peels can slow bacterial growth and inhibit toxins produced by bacteria [15–17]. To the best of our knowledge there is no study on antimicrobial activity of Golnar.

In this study, we investigated the antibacterial activity of Golnar against bacteria causing foodborne diseases, based on the traditional use of Golnar as an antibacterial agent. In addition, the total phenolic and flavonoid contents of ethanol extract and its fractions were determined.

## Methods

### Plant material

Shade dried Golnar were obtained from Darab, Fars Province, Iran in November 2012 and were identified by Dr. Asgarpanah in Department of Pharmacognosy, Pharmaceutical Sciences Branch, Islamic Azad University, Tehran, Iran. A sample was deposited in the herbarium of the university with voucher specimen No.733. Flowers were then ground down to fine powder by a mechanical miller.

### Preparation of ethanol extract

750 g of the grounded Golnar was exhaustively extracted by maceration with EtOH (3 × 1.5 lit). The extract was evaporated to yield the residue (195 g). The dried ethanol extract (EE) was kept in the refrigerator at 4 °C.

### Preparation of fractions

According to the pre-evaluation of the antibacterial effect of EE, the extract was fractioned by chloroform (C), ethyl acetate (EA), methanol (M) and water (W), successively. The obtained fractions were filtered through paper filter Whatman No. 1 to remove the solid particles and then concentrated on rotary evaporator. The samples were then stored at 4 °C.

### Bacterial strains

Six different microorganisms including *Staphylococcus aureus* ATCC 25923, *Bacillus cereus* PTCC 1247, *Listeria monocytogenes* ATCC 7644, *Escherichia coli* ATCC 25922, *Shigella dysantriae* PTCC 1188, and *Salmonella typhi* ATCC 19430 were used for evaluation of antibacterial effects of the extract and the fractions. The microorganisms were obtained from Iranian Research Organization for Science and Technology, Persian Type Culture Collection (PTCC), Tehran, Iran. The microorganisms were cultured on Mueller Hinton Agar (MHA) medium (Merck, Germany) and incubated at 37 °C for 24 h.

### Antibacterial susceptibility test

The agar well-diffusion method was conducted for primary evaluation of the inhibitory effects of EE and its fractions against test microorganisms [18, 19]. The Muller Hinton agar (MHA) medium was purchased from Merck Company, Germany. The wells (6 mm diameter) were made in the medium which was streaked with a suspension of the microorganism in saline with a turbidity equivalent to a 0.5 McFarland standard.

The extract and the fractions were serially diluted from 500 to 1.95 mg/ml by sterile Tween 20 (20 % v/v). 100 μl of different concentrations of C, EA, M, W fractions, EE extract, and solvent (as negative control) were added to each well on MHA medium. The plates were then incubated overnight at 37 °C and the zones of inhibition were measured. The test was repeated three times and the means of the results were reported.

### Minimum inhibitory concentration (MIC)

The MIC of the extract and its fractions were determined by macro broth dilution method according to CLSI (Clinical Laboratory Standardization Institute) [19, 20]. The inoculums with a turbidity equivalent to 0.5 McFarland standard (1.5× 10^8^ cfu/ml) were prepared by making a direct broth suspension of isolated colonies selected from 24 h cultured bacteria on MHA plate.

Serial dilutions of ethanol extract and its fractions were prepared volumetrically in sterile tubes using Muller Hinton Broth (MHB) medium (Merck, Germany). The 0.5 McFarland suspensions were diluted by MHB (1:150). One ml of these adjusted inoculums were added to each of the tubes containing 1 ml of dilutions of the ethanol extract or its fractions. Therefore, the final inoculums were 5 × 10^5^ CFU/ml. The tubes were incubated for 24 h at 37 °C, and then evaluated for bacterial growth. The lowest concentration with no visible growth was considered as minimum inhibitory concentrations (MICs) of the extract and the fractions [20].

### Minimum bactericidal concentration (MBC)

To confirm MICs and to establish MBC, 50 μl of each tube with no visible growth was removed and inoculated in MHA plates. After 48 h of aerobic incubation at 37 °C, the numbers of surviving microorganisms were determined. MBC was defined as the lowest concentration at which no growth of bacteria was seen [20]. Each experiment was repeated at least three times.

### Total phenolic content

Folin Ciocalteu reagent was used for the analysis of total phenolic content of the extract and the fractions [3]. Stock solutions of the ethanol extract and the fractions in methanol (10 mg/ml) were prepared and 0.02 ml of each stock solution was added to 1.58 ml of distilled water in a test tube. Then, 0.1 ml of diluted Folin Ciocalteau reagent was added to the test tube. The mixture was kept at room temperature for 3 min and then, 0.3 ml Na_2_CO_3_ 7.5 % solution was added. After 30 min, absorbance of the mixture was measured at 765 nm by UV-spectrophotometer (Multispec-1501 Shimadzu). A standard curve was prepared using gallic acid (Merck, Germany). The determinations were carried out in triplicate and the total phenolic content was expressed as gallic acid equivalents (mg of GAE/g of sample) [3].

### Total flavonoid content

The total flavonoid contents were measured by a colorimetric assay [21, 22]. The dried extract was dissolved in 80 % methanol to obtain a final concentration of 1 mg/ml. The calibration curve was prepared using 0.1–1 ml aliquots of Rutin solution, 500 μL of the acetic acid solution, 2 ml of the pyridine solution and 1 ml of the aluminum chloride solution. The final volume was adjusted to 10 ml with 80 % methanol and the final Rutin concentration was 1–10 μg/ml. To quantify the flavonoids, 0.5 ml of the ethanol extract or the fractions was transferred to a test tube and 0.5 ml of the acetic acid solution, 2 ml of the pyridine solution, 1 ml of the reagent aluminium chloride solution and 6 ml of 80 % methanol were added. The samples were kept at room temperature for 30 min and the absorbances of the mixtures were measured in 420 nm. The test was performed three times and the flavonoid content was expressed as milligrams of Rutin equivalents (RE) per gram of sample of extracts (mg RE/g) [21, 22].

### Statistical analysis

Data were presented as mean ± SD in all tables. One-way ANOVA and Tukey’s post test were used to compare the total phenolic or flavonoid content of the ethanol extract and the fractions. Graphpad Prism 5.0 (GraphPad Software, Inc., CA, USA) was used for statistical analysis. In all experiments a value of *P* < 0.05 was considered significant.

## Results

### Yield of the fractionation

The fractionation yields are shown in Table 1.

**Table 1:** The yield of fractions from Golnar ethanol extract

Type of fractions	Yield %
Methanol	25
Water	25
Ethyl acetate	10
Chloroform	10

### Evaluation of antibacterial effect by the Agar Diffusion Method

The diameter of inhibition zones made by the ethanol extract (EE) and its fractions (C, EA, M, W) are shown in Table 2. Tween 20 (20 % V/V), as negative control, showed no inhibitory effect against the tested strains. As shown in Table 2, the ethanol extract and its fractions showed antibacterial activity against all of the tested microorganisms (diameters of zone of inhibition ranging between 10 and 34 mm). The largest inhibition zone made by TE extract and its fractions was against *S. aureus*. The methanol and water fractions reflected larger inhibition zones while ethyl acetate and chloroform fractions showed smaller zones.

**Table 2:** Mean of Growth inhibition zone diameter (mm) of ethanol extract of Golnar and its fractions (*n = 3*)

Extract concentration		500 mg/ml	250 mg/ml	125 mg/ml	62.5 mg/ml	31.25 mg/ml	15.62 mg/ml	7.8 mg/ml	3.9 mg/ml	1.95 mg/ml
Microorganism										
*Staphylococcus aureus* ATCC 25923	M	32 ± 0	30 ± 0.6	29 ± 1	28 ± 1	27 ± 0	26 ± 0.6	25 ± 0.6	24 ± 1	23 ± 0
	W	34 ± 0.6	32 ± 1	31 ± 1	30 ± 0.6	28 ± 1	27 ± 0.6	26 ± 1	25 ± 0.6	24 ± 0.6
	EA	31 ± 1	28 ± 1	27 ± 1	26 ± 0.6	24 ± 1	22 ± 0	18 ± 0	15 ± 1	13 ± 0
	C	19 ± 1	16 ± 0	14 ± 0.6	13 ± 0.6	-	-	-	-	-
	EE	32 ± 0	30 ± 0	28 ± 0.6	27 ± 1	25 ± 1	24 ± 1	23 ± 1	22 ± 0	21 ± 0.6
*Bacillus cereus* PTCC 1247	M	29 ± 0	21 ± 0	19 ± 1	17 ± 1	16 ± 1	15 ± 0.6	14 ± 0.6	13 ± 0.6	12 ± 0.6
	W	28 ± 1	23 ± 1	22 ± 0	19 ± 0	18 ± 0	16 ± 0	14 ± 0.6	13 ± 0.6	12 ± 0.6
	EA	29 ± 0.6	23 ± 0.6	20 ± 0.6	14 ± 1	13 ± 1	12 ± 0	11 ± 0	10 ± 0	-
	C	17 ± 0	13 ± 1	12 ± 1	11 ± 1	10 ± 0.6	-	-	-	-
	EE	28 ± 0.6	25 ± 0.6	23 ± 0	19 ± 0	18 ± 1	16 ± 1	15 ± 1	12 ± 0	11 ± 0
*Listeria monocytogenes* ATCC 7644	M	30 ± 0	26 ± 1	22 ± 0.6	19 ± 0.6	17 ± 0.6	15 ± 0	14 ± 0	11 ± 0	10 ± 0.6
	W	30 ± 0.6	25 ± 1	23 ± 1	20 ± 0	17 ± 0	15 ± 0	14 ± 0.6	12 ± 0.6	11 ± 0.6
	EA	28 ± 0	22 ± 0	20 ± 1	17 ± 0.6	13 ± 1	-	-	-	-
	C	18 ± 1	14 ± 1	11 ± 0.6	-	-	-	-	-	-
	EE	32 ± 1	28 ± 1	26 ± 1	26 ± 0	22 ± 0	18 ± 0	14 ± 0.6	13 ± 0.6	11 ± 0.6
*Shigella dysantriae* PTCC 1188	M	32 ± 0.6	29 ± 0.6	28 ± 0.6	28 ± 0	27 ± 0	26 ± 1	25 ± 1	24 ± 0	23 ± 0.6
	W	30 ± 1	28 ± 0.6	27 ± 0.6	26 ± 0	25 ± 0	24 ± 0	22 ± 0.6	20 ± 1	19 ± 1
	EA	30 ± 0	27 ± 0	24 ± 0.6	21 ± 0.6	20 ± 0.6	19 ± 0	17 ± 0	15 ± 0.6	12 ± 0.6
	C	18 ± 0.6	15 ± 1	13 ± 1	12 ± 1	-	-	-	-	-
	EE	30 ± 1	27 ± 0	25 ± 0	24 ± 0.6	23 ± 1	21 ± 1	20 ± 1	19 ± 0	18 ± 0
*Salmonella typhi* ATCC 19430	M	31 ± 0.6	24 ± 0	21 ± 0	18 ± 0.6	14 ± 0.6	13 ± 0.6	12 ± 0.6	11 ± 0.6	10 ± 0
	W	30 ± 0	25 ± 0	22 ± 0	16 ± 0.6	13 ± 0.6	12 ± 0.6	10 ± 0.6	-	-
	EA	23 ± 0.6	19 ± 1	15 ± 1	12 ± 1	11 ± 1	-	-	-	-
	C	14 ± 1	10 ± 1	-	-	-	-	-	-	-
	EE	27 ± 0.6	24 ± 0	22 ± 0	21 ± 0	17 ± 0	14 ± 0	11 ± 0.6	-	-
*Escherichia coli* ATCC 25922	M	28 ± 1	19 ± 1	15 ± 0.6	11 ± 0	10 ± 0	-	-	-	-
	W	25 ± 0.6	15 ± 0.6	12 ± 0	10 ± 0	-	-	-	-	-
	EA	23 ± 1	18 ± 1	15 ± 1	12 ± 0.6	-	-	-	-	-
	C	12 ± 0	-	-	-	-	-	-	-	-
	EE	22 ± 1	15 ± 0.6	13 ± 0.6	10 ± 0	-	-	-	-	-

M, W, EA, C, and EE represents methanol, water, ethyl acetate fractions and ethanol extract, respectively, Zone of inhibition, including the diameter of the well (6 mm); mean ± SD value of three independent experiments

### Determination of MIC

Table 3 shows MIC values of the extract and the fractions against all tested microorganism. The MIC values obtained in this study ranged from 0.19 to 12.5 mg/ml. The ethanol extracts and the methanol and water fractions showed the lowest MIC (0.19 mg/ml) against *S. aureus*. This indicates the highest sensitivity of *S. aureus* among the six microorganisms tested in this study*. E. coli* was the most resistant microorganism against all of the fractions (MIC equal to 12.5 mg/ml for the chloroform fraction and 3.12 mg/ml for the water and the methanol fractions).

**Table 3:** Minimum inhibitory concentration (mg/ml) of ethanol extract of Golnar and its fractions (*n = 3*)

Extract and fractions	M mg/ml	W mg/ml	EA mg/ml	C mg/ml	EE mg/ml
Microorganism					
*Staphylococcus aureus* ATCC 25923	0.19	0.19	0.39	3.12	0.19
*Bacillus cereus* PTCC 1247	0.19	0.19	0.39	3.12	0.39
*Listeria monocytogenes* ATCC 7644	1.56	3.12	3.12	6.25	6.25
*Escherichia coli* ATCC 25922	3.12	3.12	6.25	12.5	6.25
*Shigella dysantriae* PTCC 1188	0.39	0.39	0.39	6.25	0.39
*Salmonella typhi* ATCC 19430	1.56	3.12	3.12	6.25	6.25

M, W, EA, C, and EE represents methanol, water, ethyl acetate fractions and ethanol extract, respectively, all tests were done in triplicate

### Determination of MBC

MBC of the ethanol extract and the fractions are shown in Table 4. The MBC values obtained in this study ranged from 0.78 to 50 mg/ml. The lowest MBC value of 0.78 mg/ml was observed from EE extract against *S. aureus* which reflects the highest sensitivity of *S. aureus* among the six microorganisms tested in this study*.* Similar to results of MIC determination, *E. coli* was the most resistant microorganism against all the fractions and the extract (MBC equal to 50 mg/ml for chloroform fraction). It may be because of the differences in their cell wall composition and presence of second membrane in Gram negative bacteria cellwall.

**Table 4:** Minimum bactericidal concentration (mg/ml) of ethanol extract of Golnar and its fractions (*n = 3*)

Extract and fractions	M mg/ml	W mg/ml	EA mg/ml	C mg/ml	EE mg/ml
Microorganism					
*Staphylococcus aureus* ATCC 25923	1.56	1.56	3.12	12.50	0.78
*Bacillus cereus* PTCC 1247	1.56	1.56	3.12	12.50	1.56
*Listeria monocytogenes* ATCC 7644	6.25	6.25	12.50	12.50	25.00
*Escherichia coli* ATCC 25922	12.50	12.50	25.00	50.00	25.00
*Shigella dysantriae* PTCC 1188	3.12	3.12	3.12	25.00	1.56
*Salmonella typhi* ATCC 19430	6.25	12.50	12.50	25.00	25.00

M, W, EA, C, and EE are represents methanol, water, ethyl acetate fractions and ethanol extract, respectively; all tests were done in triplicate

### Total phenolic content

After making a standard calibration curve by gallic acid (y = 1.48× –0.05, *r*^2^ = 0.99), the total phenolic content of the extract and the fractions were measured. The total phenolic content was ranged from 3.8 to 18.1 mg GAE/g of dry powder. As shown in Table 5, the chloroform fraction (C) had the lowest and the methanol fraction (M) had the highest total phenolic contents.

**Table 5:** Total phenolic and flavonoid of the ethanol extract of Golnar and its fractions (*n = 3*)

Extract and fractions	Phenolic content (mg GAE/g)	Flavonoid content (mg Rutin/g)
Water	17.8 ± 1.3^ac^	2.6 ± 0.35^hig^
Methanol	18.1 ± 1.5^a^	3.3 ± 0.14^bk^
Ethanol extract	17.6 ± 2.3^c^	1.9 ± 0.3^f^
Ethyl acetate	8.2 ± 2.14^bdj^	1.3 ± 0.14^ad^
Chloroform	3.8 ± 0.9^bdej^	1.1 ± 0.12^hdf^

Data were shown as mean ± SD

^*a*^ *p* > 0.05, ^*b*^ *p* < 0.001, ^h^ *p* < 0.05 difference compared with the ethanol extract

^*c*^ *p* > 0.05, ^*d*^ *p* < 0.001, ^i^ *p* < 0.05 difference compared with the methanol fraction

^*e*^ *p* < 0.001, ^f^ *p* > 0.05 difference compared with the ethyl acetate fraction

^g^ *p* < 0.001 difference compared with the chloroform fraction

^j^ *p* < 0.001, ^k^ *p* < 0.05 difference compared with the water fraction

### Total flavonoid content

Standard calibration curve of Rutin was used to evaluate the content of flavonoid in the extract and the fractions (y = 0.02× –0.05, *r*^*2*^ = 0.97). Results are shown in Table 5. The lowest flavonoid content was seen in the chloroform fraction (1.1 mg RE/g of dry powder) and the highest was seen in the methanol fraction (3.3 mg RE/g of dry powder).

## Discussion

Golnar has been used in traditional Iranian medicine for healing wounds, treating large intestine ulcers, strengthening gums, and the treatment of diarrhea and oral infections [9–11]. In this study, we evaluated the antibacterial effects of the ethanol extract of Golnar and its fractions against bacterial strains that cause food poisoning. Among the bacteria used in this study, *E. coli* is the most common cause of diarrhea in developing countries [23]. The second most common cause of bacterial foodborne diseases in the United States is *Salmonella. Shigella dysantriae* is the third important microorganism involved in food and water contamination [24]. Other bacteria such as *S. aureus* and *B. cereus* are also involved in food poisoning [25, 26]. *L. monocytogenes* is a Gram-positive bacterium responsible for the severe foodborne illness, listeriosis. This disease is primarily transmitted through various foods such as fish, dairy products, cured or processed meat, egg, poultry, seafood, salad, fruits and vegetables [27]. Listeriosis is a severe infection and has been associated with a mortality rate as high as 30–40 % [28]. Using synthetic preservatives for prevention and antibiotics for treatment of foodborne diseases may result to several unpleasant effects including hypersensitivity, immune-suppression and allergic reactions [29]. Therefore, there is an increasing need to develop new alternative natural agents as preservative or antibacterial agents [30].

In this study, the antibacterial activity of the ethanol extract and its fractions were primarily evaluated by the agar well diffusion method. The results indicated a broad spectrum activity against both gram positive and gram negative bacteria. The largest inhibition zone (34 mm) was seen with the water fraction for *S. aureus* at the concentration of 500 mg/ml. The smallest inhibition zone at this concentration was seen for *E. coli* with the diameter of 12 mm related to the chloroform fraction. The results showed that *S. aureus* (Gram positive bacteria) and *Shigella dysantriae* (Gram negative bacteria) could be more sensitive based on their larger inhibition zones.

All fractions and the ethanol extract showed inhibitory effects against *S. aureus* at a concentration equal to 1.95 mg/ml and higher. However, the chloroform fraction showed inhibitory effect at 62.5 mg/ml and higher concentrations. This profile of the antibacterial effect of the ethanol extract and the fractions were confirmed by determining the MICs and MBCs. In fact, the polar fractions generally showed better antibacterial activity, which can be related to the total phenolic content of the fractions. Polyphenols are hydrophilic phytochemicals and hydrophilic solvents are more effective agents for the extraction [31, 32]. The total phenolic content of the extract and its fractions were expressed in term of gallic acid equivalent. According to the results, EE contains 17.6 mg GAE/g of phenolic content, while methanol and water fractions contain 18.1 mg GAE/g and 17.8 mg GAE/g, respectively. The ethyl acetate and chloroform fractions contain only 8.2 mg GAE/g and 3.8 mg GAE/g of phenolic contents, respectively. Moreover, one-way ANOVA followed by Tukey’s test revealed a significant decrease in phenolic content in the ethyl acetate and chloroform fractions compared to the water fraction (*P <* 0.001), methanol fraction (*P <* 0.001), and ethanol extract (*P <* 0.001). The antimicrobial activities of phenolic compounds have been demonstrated in previous studies [31, 33]. Our results are in agreement with the previous studies on pomegranate (*P. granatum* fruit) [17]. Although chemical components of Golnar were not analyzed in this study, however, it could be suggested that the phenolic compounds are involved in the antibacterial effects we reported.

Quantitative evaluations of antimicrobial activity were done against test microorganisms using the broth dilution method. Considering the MICs, the best results were related to EE extract, as well as M and W fractions (0.19 mg/ml) against *S. aureus*. Gram negative bacteria were more resistant to the extract. Presence of an outer membrane in Gram negative bacteria can explain the resistance. *E. coli* was found to be the most resistant bacteria with the MIC of 12.5 mg/ml for the most effective fraction and 50 mg/ml for the least effective fraction. Al-zoreky has reported that the 80 % methanol extract of pomegranate fruit peels has similar effects on Gram positive and Gram negative bacteria [2].

The highest flavonoid content was measured in the methanol fraction (*P <* 0.001 and *P <* 0.05 compared with the ethanol extract and the water fraction, respectively), in which the most antibacterial action was also observed. It has been shown that the antimicrobial efficacy of the herbal extracts correlates with their flavonoid contents [34]. In addition, many flavonoids have also shown anti-infective effects through making complexes with different proteins inside the bacterial cell walls or extracellular proteins [34, 35] Such a relation between antibacterial effects and flavonoid content was suggested from the results of this study.

It has been shown that pomegranate pericarp extract enhances the antibacterial activity of ciprofloxacin against extended-spectrum beta-lactamase and metallo-beta-lactamase producing Gram-negative bacilli [3]. Considering the antibacterial effects of Golnar, there is a potential benefit in using the extract in combination with classic antibacterial agents to improve the antibacterial effects and consequently reduce the side effects of the agents.

## Conclusion

From the results of the present study, it could be concluded that the methanol and the water fractions have similar effects on Gram positive bacteria. Antibacterial effects of the methanol and water fractions on Gram negative bacteria are also relatively similar except for the methanol extract which revealed to be more effective on *Salmonella typhi*. The ethyl acetate and chloroform fractions have less effect on microorganisms, suggesting that the active antibacterial agents are most concentrated in polar fractions. The results of this study complies the traditional use of Golnar as an antibacterial agent against foodborne diseases. Moreover, there is potential for its use in the food industry and in medicine as a preservative. Clearly, further studies are necessary to evaluate the efficacy of the extract in different indications.

## References

[1] Scallan E, Hoekstra RM, Angulo FJ, Tauxe RV, Widdowson MA, Roy SL, Jones JL, Griffin PM (2011). Foodborne illness acquired in the United States—major pathogens. Emerg Infect Dis.

[2] Al-Zoreky NS (2009). Antimicrobial activity of pomegranate (*Punica grana*tum L.) fruit peels. Int J Food Microbiol.

[3] Dey D, Debnath S, Hazra S, Ghosh S, Ray R, Hazra B (2012). Pomegranate pericarp extract enhances the antibacterial activity of ciprofloxacin against extended-spectrum β-lactamase (ESBL) and metallo-β-lactamase (MBL) producing Gram-negative bacilli. Food Chem Toxicol.

[4] Naz S, Siddiqi R, Ahmad S, Rasool SA, Sayeed SA (2007). Antibacterial activity directed isolation of compounds from *Punica granatum*. J Food Sci.

[5] Babaa SA, Malikb SA (2014). Evaluation of antioxidant and antibacterial activity of methanolic extracts of *Gentiana kurroo* royle. Saudi J Biol Sci.

[6] Noormohammadi Z, Fasihee A, Homaee-Rashidpoor S, Sheidai M, Ghasemzadeh BS (2012). Genetic variation among Iranian pomegranates (*Punica granatum* L.) using RAPD, ISSR and SSR markers. Aust J Crop Sci.

[7] Hodgson RW (1917). The Pomegranate.

[8] Levin GM (2006). Pomegranate roads: a soviet botanist’s exile from Eden.

[9] Hosseini MM (1979). Tohfeye Hakim Momen.

[10] Avicenna H (1991). The canon of medicine (The Laws of Medicine).

[11] Khorasani MA (1992). Makhzanol advieh.

[12] Gould SWJ, Fielder MD, Kelly AF, Naughton DP (2009). Anti-microbial activities of pomegranate rind extracts: enhancement by cupric sulphate against clinical isolates of *S. aureus*, MRSA and PVL positive CA-MSSA. BMC Complement Altern Med.

[13] Hajimahmoodi M, Moghaddam G, Ranjbar AM, Khazani H, Sadeghi N, Oveisi M (2013). Total phenolic, flavonoids, tannin content and antioxidant power of some Iranian pomegranate flower cultivars (*Punica granatum* L.). Am J Plant Sci.

[14] Watson RR, Preedy VR (2010). Bioactive foods in promoting health: fruits and vegetables.

[15] Braga LC, Shupp JW, Cummings C, Jett M, Takahashi JA, Carmo LS, Cartone-Souza E (2005). Pomegranate extract inhibits *Staphylococcus aureus* growth and subsequent enterotoxin production. J Ethnopharmacol.

[16] Bialonska D, Ramnani P, Kasimsetty SG, Muntha KR, Gibson GR, Ferreira D (2010). The influence of pomegranate by-product and punicalagins on selected groups of human intestinal microbiota. Int J Food Microbiol.

[17] Choi JG, Kang OH, Lee YS, Chae HS, Oh YC, Brice OO. *In vitro* and *in vivo* antibacterial activity of *Punica granatum* peel ethanol extract against *Salmonella*. Evid Based Complement Alternat Med. 2011. doi:10.1093/ecam/nep105*.* 10.1093/ecam/nep105 PMC3137154 19687188

[18] Fazly-Bazzaz BS, Khajehkaramadin M, Shokooheizadeh HR (2005). Antibacterial activity of *Rheum ribes* extract obtained from various plant parts against clinical isolates of Gram-negative pathogens in Iran. Iran J Pharm Res.

[19] Shami AMM, Philip K, Muniandy S (2013). Synergy of antibacterial and antioxidant activities from crude extracts and peptides of selected plant mixture. BMC Complement Altern Med.

[20] Clinical and Laboratory Standards Institute (CLSI) (2009). M07-A8- methods for dilution antimicrobial susceptibility tests for bacteria that grow aerobically; approved standard-eighth edition.

[21] Meda A, Lamien CE, Romito M, Millogo J, Nacoulma OG (2005). Determination of the total phenolic, flavonoid and proline contents in burkina fasan honey, as well as their radical scavenging activity. Food Chem.

[22] Baba SA, Malik AH, Wani ZA, Mohiuddin T, Shah Z, Abbas N, Ashraf N (2015). Phytochemical analysis and antioxidant activity of different tissue types of Crocus sativus and oxidative stress alleviating potential of saffronextract in plants, bacteria, and yeast. S Afr J Bot.

[23] Qadri F, Svennerholm AM, Faruque ASG, Bradley SR (2005). Enterotoxigenic *Escherichia coli* in developing countries: epidemiology, microbiology, clinical features, treatment, and prevention. Clin Microbiol Rev.

[24] Edwards BH (1999). *Salmonella* and *Shigella* species. Clin Lab Med.

[25] Ding T, Wang J, Park MS, Hwang CA, Oh DH (2013). A probability model for enterotoxin production of Bacillus cereus as a function of pH and temperature. J Food Prot.

[26] Johler S, Tichaczek-Dischinger PS, Rau J, Sihto HM, Lehner A, Adam M, Stephan R (2013). Outbreak of Staphylococcal food poisoning due to SEA-producing *Staphylococcus aureus*. Foodborne Pathog Dis.

[27] García MT, Marínez Cañamero M, Lucas R, Ben Omar N, Pérez Pulido R, Gálvez A (2004). Inhibition of *Listeria monocytogenes* by enterocin EJ97 produced by *Enterococcus faecalis* EJ97. Int J Food Microbiol.

[28] Boland JAV, Kuhn M, Berche P, Chakraborty T, Bernal GD, Goebel W (2001). *Listeria* pathogenesis and molecular virulence determinants. Clin Microbiol Rev.

[29] Ahmad I, Mehmood Z, Mohammad F (1998). Screening of some Indian medicinal plants for their antimicrobial properties. J Ethnopharmacol.

[30] Berahou A, Auhmani A, Fdil N, Benharref A, Jana M, Gadhi CA (2007). Antibacterial activity of Quercus ilex bark’s extracts. J Ethnopharmacol.

[31] Alzoreky NS, Nakahara K (2003). Antibacterial activity of extracts from some edible plants commonly consumed in Asia. Int J Food Microbiol.

[32] Negi PS, Jayaprakasha GK (2003). Antioxidant and antibacterial activities of *Punica granatum* peel extracts. J Food Sci.

[33] Voravuthikunchai SP, Sririrak T, Limsuwan S, Supawita T, Iida T, Hond T (2005). Inhibitory effect of active compounds from *Punica granatum* pericarp on verocytotoxin production by Enterohemorrhagic *Escherichia coli* O157: H7. J Health Sci.

[34] Cowan MM (1999). Plant products as antimicrobial agents. Clin Microbiol Rev.

[35] Kilani-Jaziri S, Frachet V, Bhouri W, Ghedira K, Chekir-Ghedira L, Ronot X (2011). Flavones inhibit the proliferation of human tumor cancer cell lines by inducing apoptosis. Drug Chem Toxicol.

